# Ultrathin Sb_2_S_3_ solar cells processed by atomic-layer additive manufacturing

**DOI:** 10.1038/s43246-026-01279-7

**Published:** 2026-07-10

**Authors:** Micah Mc Naire, Sanja Pannen, Jonas Englhard, Pei-Chun Liao, Selina Kern, David Zanders, Anjana Devi, Ryan W. Crisp, Julien Bachmann

**Affiliations:** 1 Department of Chemistry and Pharmacy, Section Materials Chemistry, Chair Chemistry of Thin Film Materials, IZNF, Friedrich-Alexander-Universität Erlangen-Nürnberg, Erlangen, Germany; 2 Inorganic Materials Chemistry, Ruhr University Bochum, Bochum, Germany; 3 Leibniz Institute for Solid State and Materials Research, Dresden, Germany; 4 Chair of Materials Chemistry, Faculty of Chemistry and Food Chemistry, TU Dresden, Dresden, Germany; 5 ATLANT 3D Nanosystems ApS, Taastrup, Denmark

**Keywords:** Electronic materials, Surface patterning, Solar cells, Solid-state chemistry, Chemical engineering

## Abstract

The most conventional atomic layer processing method, atomic layer deposition (ALD), delivers ultrathin blanket coatings with sub-nanometer thickness precision. Lateral confinement underpins the direct atomic layer processing (DALP^®^) family of deposition techniques. ALD chemistry applied to DALP^®^ is a 3D printing method called atomic-layer additive manufacturing (ALAM). Here, we demonstrate the applicability of ALAM to the additive buildup of the crucial ZnS / Sb_2_S_3_ / V_2_O_5_ semiconductor stack which constitutes an functional inorganic solar cell. To this goal, ALAM processes are first optimized and evaluated for the individual materials vanadium(V) oxide, zinc sulfide, and antimony(III) sulfide. We establish the layer-by-layer growth mode controlled by self-limiting surface chemistry and characterize the materials’ structure and the smooth surface morphology of ALAM-coated areas. Finally, all three materials are 3D-printed in ALAM mode in combination with electrodes and the electron acceptor titania (TiO_2_) to form functional solar cells with a 120 nm thick Sb_2_S_3_ absorber layer. This novel fabrication of solar cells highlights the advantages of using direct patterning in the prototyping and optimization of photovoltaics in research and development.

## Abstract (web summary)

This paper demonstrates the applicability of atomic-layer additive manufacturing to the additive buildup of the crucial ZnS/Sb2S3/V2O5 semiconductor stack, which constitutes a functional inorganic solar cell.

## Introduction

Photovoltaics (PV) are a mature technology deployed at the terawatt scale. Yet the high energy and material consumption in production necessitates research to significantly reduce them. Beyond Si-based solar cells, which currently dominate the PV market with >100 µm thick devices^1–7^, second-generation solar cells enable thin film (<10 µm) architectures based on materials with higher absorption coefficients, such as CIGS or CdTe^7–12^. They allow for more flexible fabrication and applications^13–17^. However, their reliance on scarce and/or toxic materials limits their commercial breakthrough^18–20^. Therefore, third-generation solar cells emphasize sustainability as the fundamental metric rather than pure power conversion efficiency (PCE). For example, antimony(III) chalcogenides represent a promising alternative, due to their high absorption coefficients (corresponding to ideal thicknesses on the order of 0.1 µm), tunable band gap, high stability, and reasonable element abundance^20–26^. Efficiencies of up to 8% have been reported for single-junction solar cells^27^ using both solution-based^27–31^ and vacuum-based deposition methods. Recently, atomic layer deposition (ALD) has become increasingly established thanks to its precise control over film thickness^32–37^. This control becomes even more necessary the thinner each layer needs to be. However, ALD has traditionally been inefficient in precursor usage and, above all, time^38–43^. Furthermore, devices rely on patterned regions instead of blanket coatings, requiring subsequent etching or patterning processes, thereby increasing process complexity. These requirements associated with ALD-processed PV imply that prototyping and optimization tasks become intolerably time-consuming as large numbers of test cells must be manufactured with parameter variations and in statistically significant amounts.

**Fig. 1:**
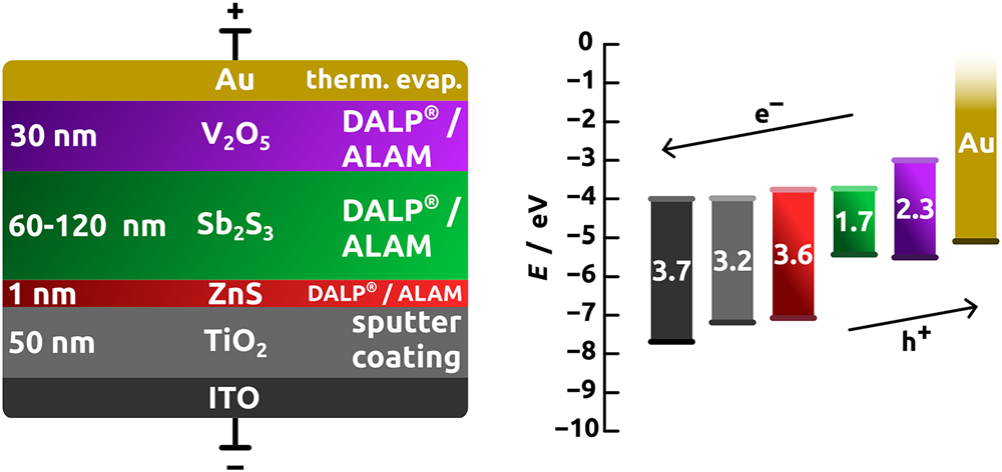
Solar cell structure. Left, semiconductor stack based on Sb_2_S_3_ light absorber layer presented in terms of its architecture and processing. Right, sketch of its electronic band structure (band gaps quoted in eV)^68–73^.

Direct atomic layer processing (DALP^®^) enables rapid generation of patterned stacks, with the additive DALP^®^ procedure atomic-layer additive manufacturing (ALAM) able to combine the layer-by-layer control of ALD with the flexibility of 3D printing^44–47^. A microfluidic gas nozzle delivers precursor gases in a laterally constrained manner (see Fig. 1a in our original publications)^47,48^, enabling localized deposition on a substrate carried by a motion stage. ALAM eliminates the need for large volumes of precursor vapors that is commensurate with traditional ALD chambers of macroscopic size. It minimizes the ALD cycle time and accelerates prototyping and optimization tasks by varying parameters and layer thicknesses on a single substrate.

Before ALAM can be applied to minimize material and energy consumption in ultrathin photovoltaics, two fundamental aspects must be established. First, ALAM processes must be developed for all semiconductors involved. Second, a geometry of the semiconductor stack and electrodes must be designed for ALAM printing. The following paragraphs establish both aspects for a PV system based on antimony sulfide (Sb_2_S_3_).

Our goal is not to deliver the world’s most efficient solar cell, and certainly not to prove economic viability of a production method. We introduce a novel prototyping method and demonstrate that a semiconductor stack can be built up in one single deposition tool—three distinct layers (ZnS, Sb_2_S_3_, V_2_O_5_) with one method and with the capability of generating several devices on one substrate. There is certainly ample room for later improvement in both the device performance and the process maturity.

## Results and discussion

### Stack design

The stack chosen for the present study relies on a thin film solar cell design (Fig. 1) that combines a crystalline ultrathin Sb_2_S_3_ intrinsic light absorber layer (variable, ~60–120 nm) with titania (TiO_2_, 50 nm) as the electron transport material and vanadium(V) oxide (V_2_O_5_, ~30 nm) as the hole transport layer^49,50^. The choice of V_2_O_5_ as the hole transport material differs from our previous studies in which spin-coated polythiophenes were used. It relies on the accessibility of V_2_O_5_ via atomic-layer processing in mild conditions, specifically, at a temperature below 200 °C and in the absence of an oxidizing agent such as ozone. An additional interfacial layer of zinc sulfide (ZnS, 1 nm) has been demonstrated to be crucial for both chemical and physical reasons^21,32,51^. This minimal stack design is well suited to demonstrate the first ALAM-printed PV device. The top gold contact is thermally evaporated through a mask, whereas commercially available transparent conducting indium tin oxide (ITO) substrates are used as the bottom contact with sputter-coated TiO_2_. Therefore, the ALAM processes for V_2_O_5_, ZnS, and Sb_2_S_3_ are established first.

### V_2_O_5_ ALAM process

**Fig. 2:**
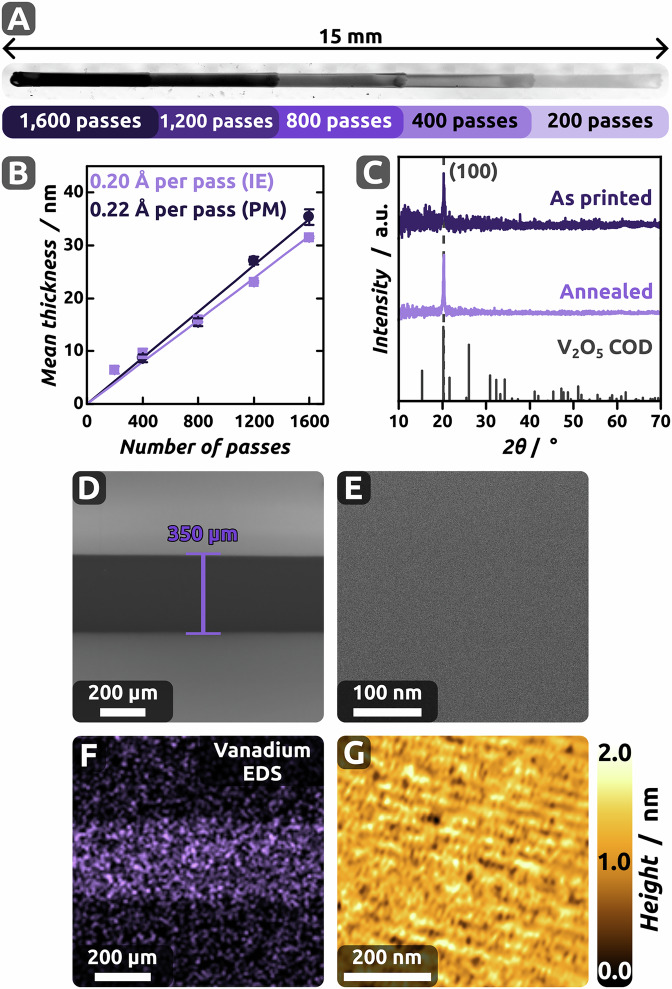
V_2_O_5_ ALAM process. **A** Optical image of segmented line print of V_2_O_5_ using ALAM. **B** Averaged growth rates as determined by imaging ellipsometry (lavender) and profilometry (deep purple) using multiple segmented line prints show ALD-type growth. The error bars represent standard deviations derived from *n* = 9 individual thickness measurements on *n*’ = 3 distinct, nominally identical samples. **C** GI-XRD confirms the presence of crystalline shcherbinaite V_2_O_5_ (COD ID 9012221). **D**, **E** SEM, **F** EDS and **G** AFM show the presence of a smooth and homogenous V_2_O_5_ thin film with roughness similar to the Si(100) substrate.

We first investigate the surface chemistry of V_2_O_5_ ALAM on native Si(100) wafers for print and material quality. We choose the metal-organic precursor vanadyl triisopropoxide (OV(O^*i*^Pr)_3_) to react with water at a substrate temperature of 150 °C. ALAM-patterned lines of 350 µm width appear with orange to blue hues depending on the number of passes performed (Fig. 2A). Lines featuring a distinct number of passes on specific segments allow for rapid determination of growth rates within a single sample using imaging ellipsometry and profilometry. The five individual thickness values presented in Fig. 2 have been deposited in less than 45 min. As seen in our previous work, ALAM retains the characteristic layer-by-layer growth of ALD^44–47^. Indeed, we find that V_2_O_5_ ALAM exhibits a linear growth rate of ~0.2 Å per pass (Fig. 2B), very similar to reports of conventional chamber-based ALD^52,53^. Scanning electron microscopy (SEM) and atomic force microscopy (AFM) analyses further show that the resulting V_2_O_5_ deposits are exceptionally smooth and homogenous with minimal to no impurities and a root-mean-square surface roughness of less than a nanometer. (Fig. 2D, E, G) Energy-dispersive X-ray microanalysis (EDS) mapping and grazing-incidence X-ray diffraction (GI-XRD) further demonstrate the existence of pure, crystalline shcherbinaite V_2_O_5_ before and after annealing at 250 °C for 2 min (Fig. 2C, F).

### ZnS ALAM process

**Fig. 3:**
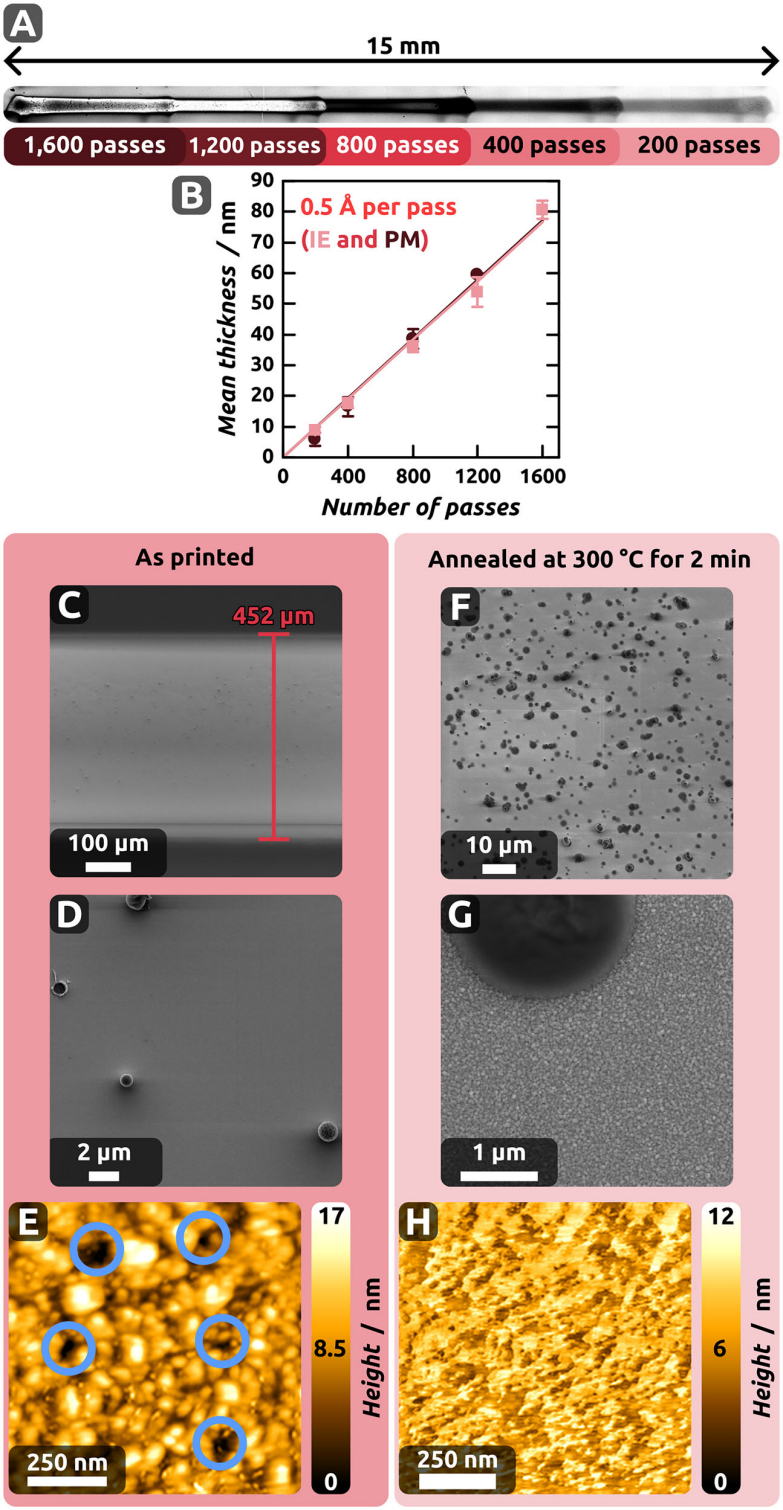
ZnS ALAM process. **A** Optical image of a segmented line print of ZnS using ALAM. **B** Averaged growth rates determined by imaging ellipsometry (salmon pink) and profilometry (dark red) using multiple segmented line prints show ALD-type growth. The error bars represent standard deviations derived from *n* = 9 individual thickness measurements on *n*’ = 3 distinct, nominally identical samples. The as-printed thin film (**C**–**E**) shows a smooth base layer with discernable pinholes and agglomerates on top. **F**–**H** Upon annealing at 300 °C for 2 min the pinholes are eliminated with an improved surfaces roughness, however the thin film still features agglomerates on top.

For ZnS, we apply the metal-organic precursor bis(3-dimethylaminopropyl)zinc, Zn(DMP)_2_, with H_2_S as the reactant. This alternative to the classical diethylzinc^54–57^ features lower vapor pressure, lower reactivity, and non-pyrophoric behavior. It is therefore inherently safer with improved material economy^58^. Only the very sparing use of precursor vapor by ALAM renders this particular compound suitable for deposition^46^. The well-behaved chemistry enabled by Zn(DMP)_2_ allows for printing very defined 450-µm wide lines with a linear growth rate of 0.5 Å per pass, comparable to reported chamber-based ALD processes, indicating that the growth mode is controlled by surface chemistry (Fig. 3)^56,57^. SEM indicates the presence of smooth and homogenous ZnS thin films with additional agglomerates. AFM exhibits an increased roughness of ~2.6 nm and reveals smaller pinholes in a 50 nm thick film. Annealing the ZnS films at 300 °C under N_2_ for 2 min eliminates pinholes and improves the surface roughness to 1.8 nm though the agglomerates are still present.

**Fig. 4:**
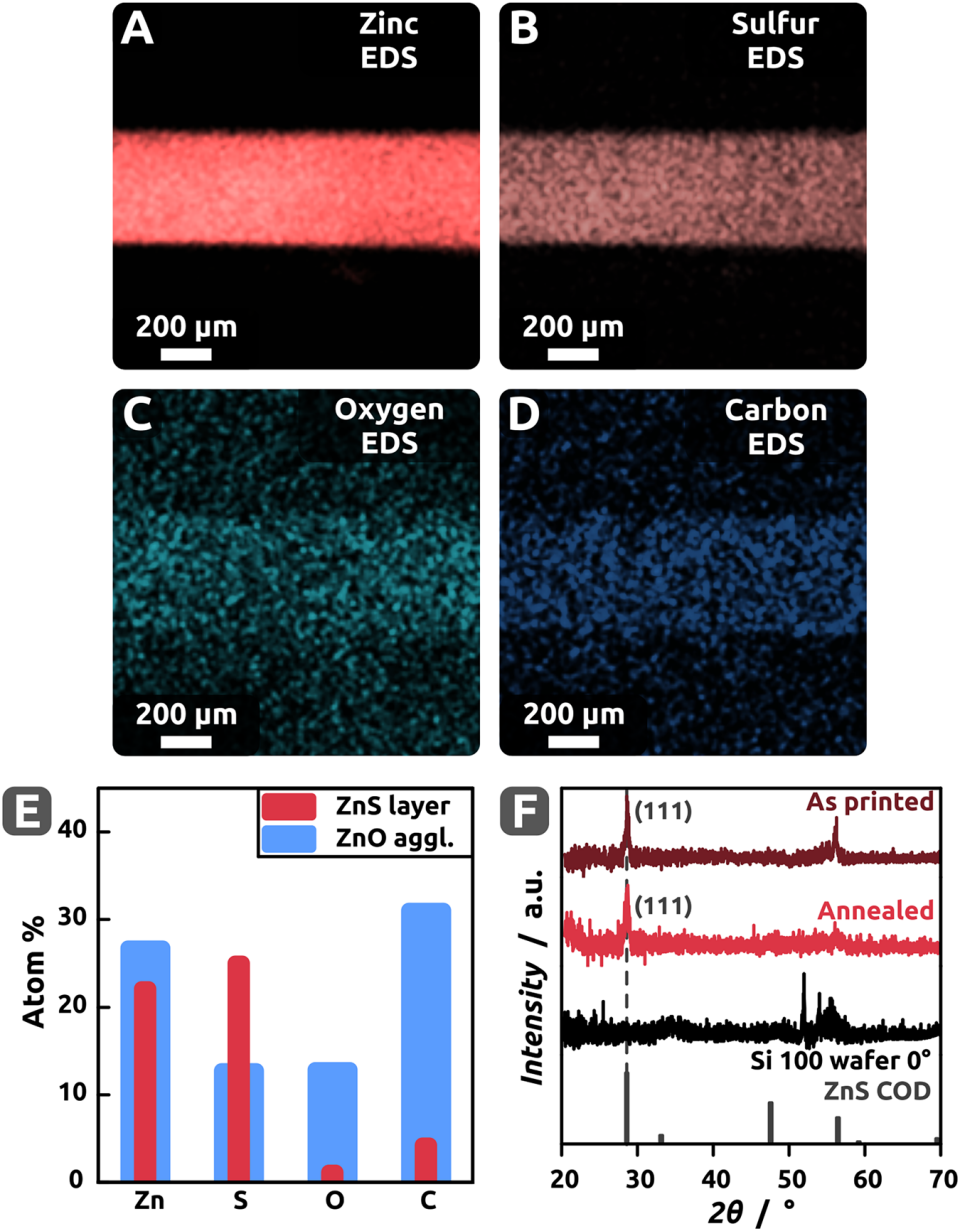
ZnS ALAM material. **A**–**D** EDS mappings of the printed ZnS lines show a stoichiometric ZnS deposit with carbon and oxygen impurities. EDS quantifications (**E**) of the underlying ZnS film and the agglomerates confirm the purity of the ZnS thin film in contrast to the agglomerates on top of it, which are dominated by ZnO caused by precursor accumulation and decomposition. **F** GI-XRD measurements demonstrate the presence of crystalline ZnS (sphalerite, COD ID 9000107).

EDS (Fig. 4A–D) characterizes a rather compositionally pure ZnS base layer, which is further supported by subsequent GI-XRD (Fig. 4F), but with high carbon and oxygen contents in the agglomerates (Fig. 4E). This is strongly indicative of unreacted Zn(DMP)_2_ precursor remnants, which are subsequently decomposed upon exposure to air. These agglomerates will inevitably have a negative impact on solar cell performance, and we assume that they can be avoided by running the ZnS process at a temperature higher than the current limitation of our ALAM prototype. This will involve additional thermal management between the sample heater and the motion stage.

### Sb_2_S_3_ ALAM process

**Fig. 5:**
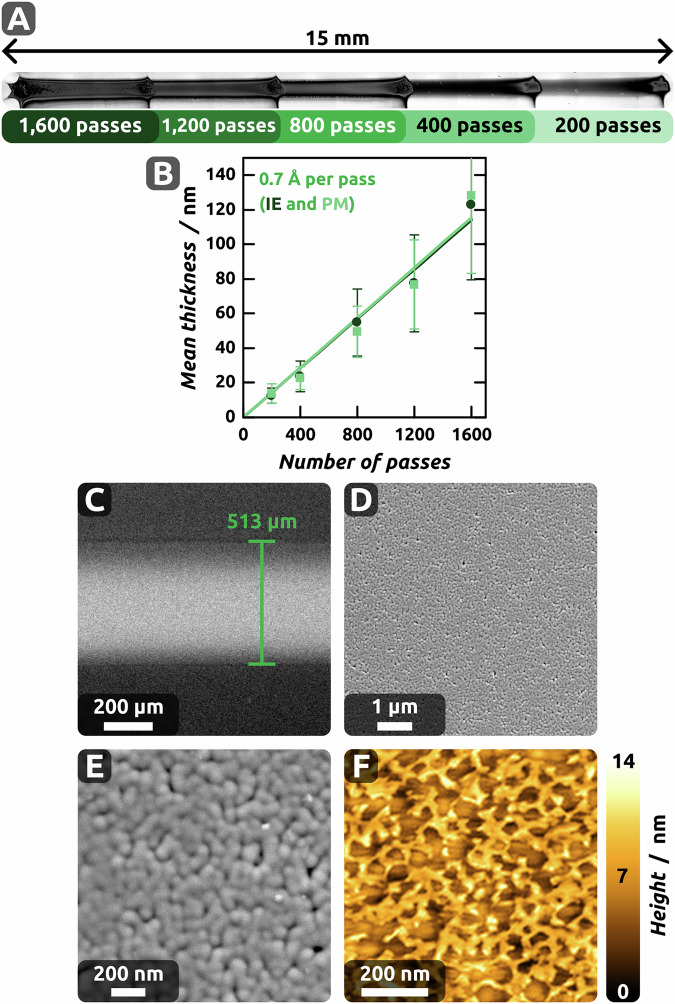
Sb_2_S_3_ ALAM process. **A** Optical image of segmented line print of Sb_2_S_3_ using ALAM. **B** Averaged growth rates determined by imaging ellipsometry (pale moss) and profilometry (forest green) using multiple segmented line prints show ALD-type growth. The error bars represent standard deviations derived from *n* = 9 individual thickness measurements on *n*’ = 3 distinct, nominally identical samples. The as-printed thin films (**C**–**F**) exhibit visible pinholes.

The Sb_2_S_3_ light absorber layer is printed using tris(dimethylamido)antimony(III), (Me_2_N)_3_Sb, and H_2_S. While in conventional chamber-based ALD (Me_2_N)_3_Sb is usually volatilized at elevated temperature, in ALAM mode it necessitates accurate cooling to –3 °C. In these conditions, ALAM yields accurate prints of thin Sb_2_S_3_ lines with slightly increased 500-µm width (Fig. 5A). Growth is linear with ALD-similar 0.7 Å per pass (Fig. 5B)^33,36,59,60^. When printed on Si substrates (with a native SiO_2_ layer) in the absence of a ZnS adhesion layer, thin Sb_2_S_3_ films exhibit discontinuities as deposited (Fig. 5C–F).

**Fig. 6:**
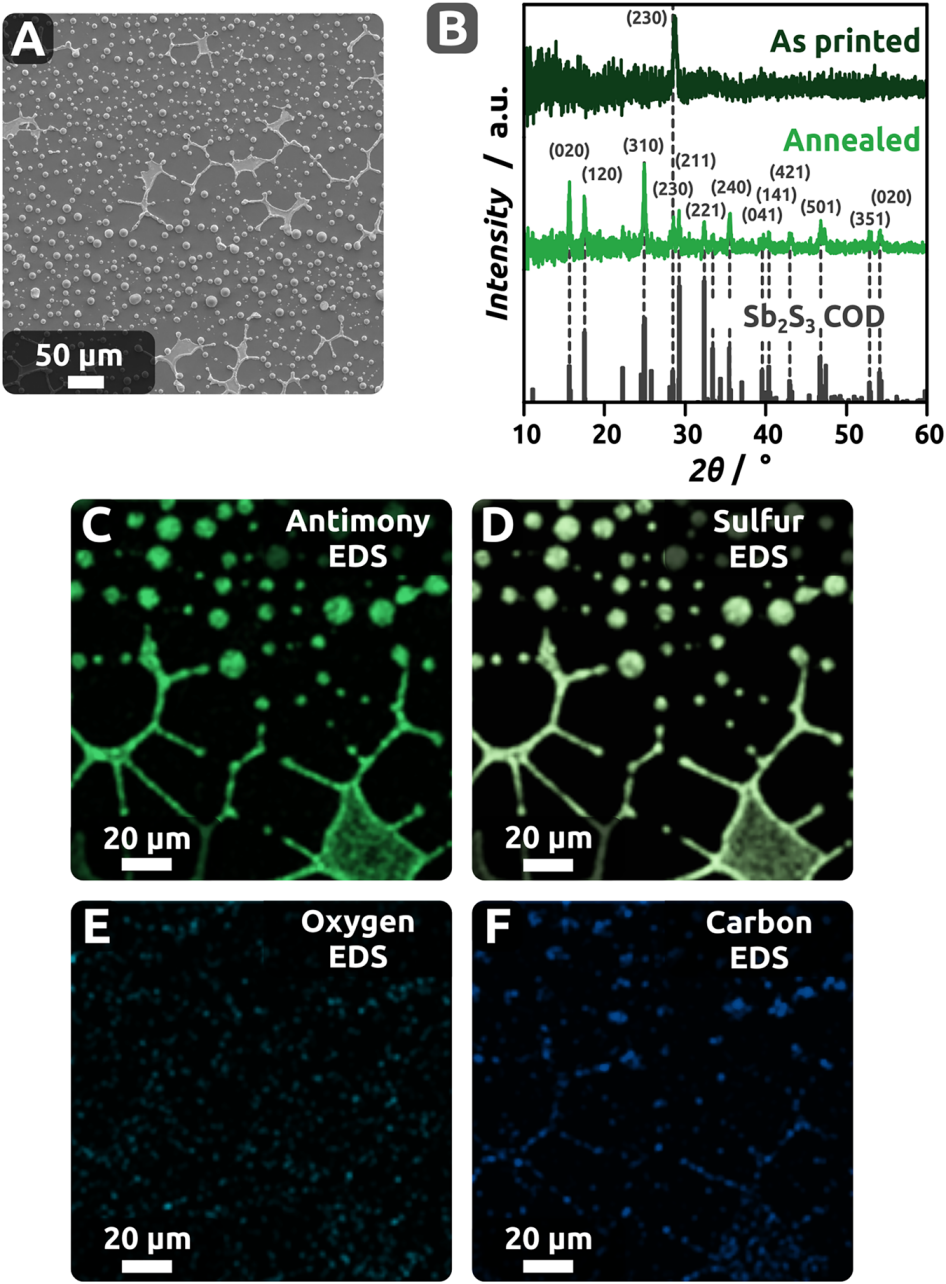
Sb_2_S_3_ dewetting on metal oxides. **A** SEM measurements of the annealed Sb_2_S_3_ printed thin film shows strong dewetting on the Si(100) wafer. **B** GI-XRD measurements and **C**–**F** EDS mapping shows highly pure, crystalline Sb_2_S_3_ (stibnite, COD ID 9003460).

Sb_2_S_3_ is deposited at 180 °C in its well-known orange, amorphous form. Crystallizing it to its electrically conductive, black stibnite structure involves annealing in conditions for which there is ample literature precedents. We have demonstrated in the past that annealing a-Sb_2_S_3_ as a continuous thin film on planar ZnS-coated substrates maintains the continuity and yields laterally large (micron-sized) crystals while maintaining the low roughness of the layer^21,34,61,62^. Upon annealing at 300 °C for 2 min, our ALAM-deposition Sb_2_S_3_ films (Fig. 6) exhibit complete dewetting on the native Si(100) wafer, as reported^21^. Nevertheless, EDS mapping and GI-XRD show that the solid consists of pure, crystalline Sb_2_S_3_.

**Fig. 7:**
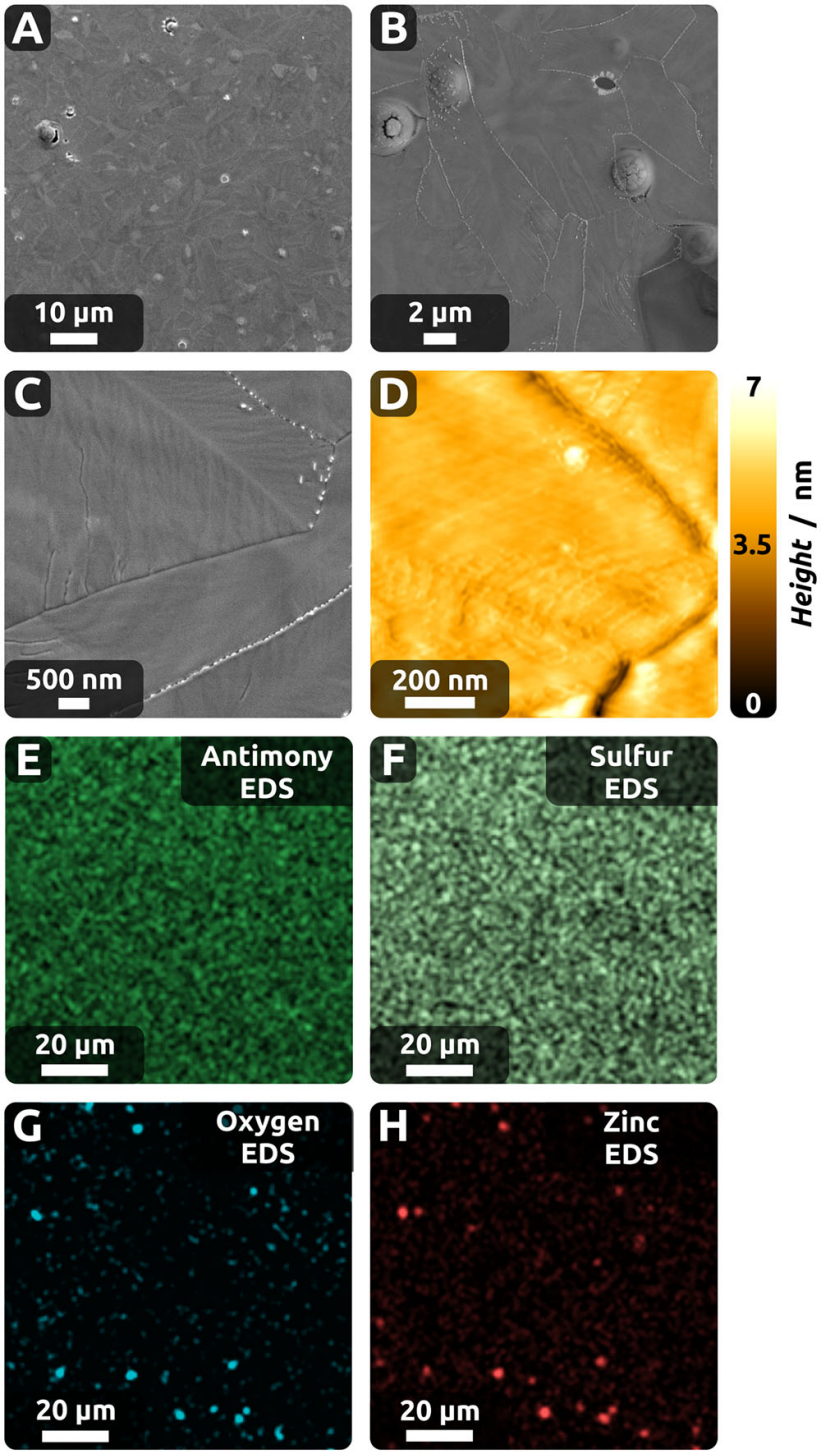
Sb_2_S_3_ annealing to a continuous, crystalline film. SEM (**A**–**C**) and AFM (**D**) measurements of the annealed Sb_2_S_3_ thin film with a ~1 nm thin ZnS adhesion layer (on Si(100) substrate) reveal a smooth and homogenous surface morphology. **E**–**H** EDS mapping confirms the presence of ZnO-rich agglomerates underneath the pristine Sb_2_S_3_ thin film already observed in Fig. 4.

In contrast to this, depositing the same Sb_2_S_3_ onto a Si(100) wafer preliminarily coated with only 1 nm of ALAM-printed ZnS yields after annealing an exceptionally smooth (AFM roughness <1 nm), crystalline and closed thin film with discernible grain boundaries, as seen in SEM (Fig. 7A–D). This observation is fully parallel to our earlier studies using conventional chamber-based ALD^21,51^. Of course, the underlying ZnO-rich agglomerates are still present underneath the pristine Sb_2_S_3_ thin film.

### Solar cell design

Each material of the solar cell stack printed by ALAM has sufficient thin film quality and control to allow fabrication of miniaturized functional devices that require careful consideration of the lateral geometry for the electrical contacts and the semiconductors. Inadequate fitting of the metallic contacts to the diminutive ALAM feature sizes would generate short-circuits.

**Fig. 8:**
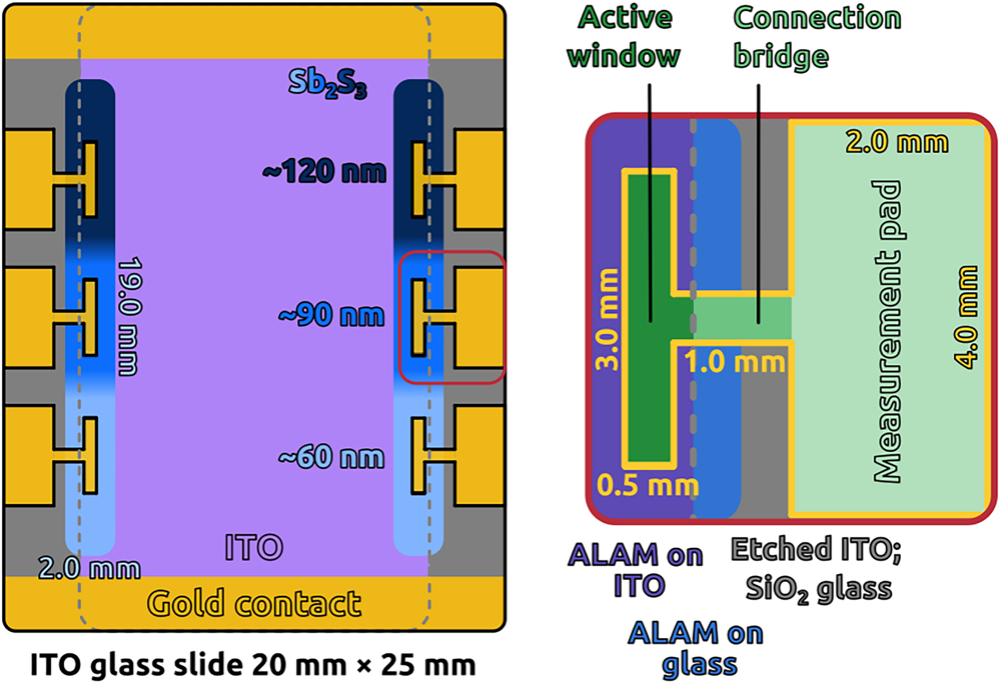
Schematic of the ALAM printed solar cell design with subsequently evaporated gold contacts (golden color in the overview left) defined by masking. This design allows for the fabrication of six solar cells with up to six different configurations. It minimizes the risk of short-circuiting between the top gold contacts and the conductive ITO bottom electrode by judicious placement of a chemically etched ITO-free surface on either side of the substrate (gray).

Before attempting to generate large numbers of cells on one substrate for rapid prototyping, we establish a proof of principle design of the contacts that places six individual cells on one substrate (Fig. 8). We define an active window size of 0.5 mm × 3 mm. The gold connection bridge leading to the measurement pad is positioned over a preliminarily etched non-conductive glass sideline (gray surface in Fig. 8), with the solar active materials slightly overlapping. This approach necessitates sufficient accuracy in the alignment of ALAM prints and subsequent gold evaporation.

**Fig. 9:**
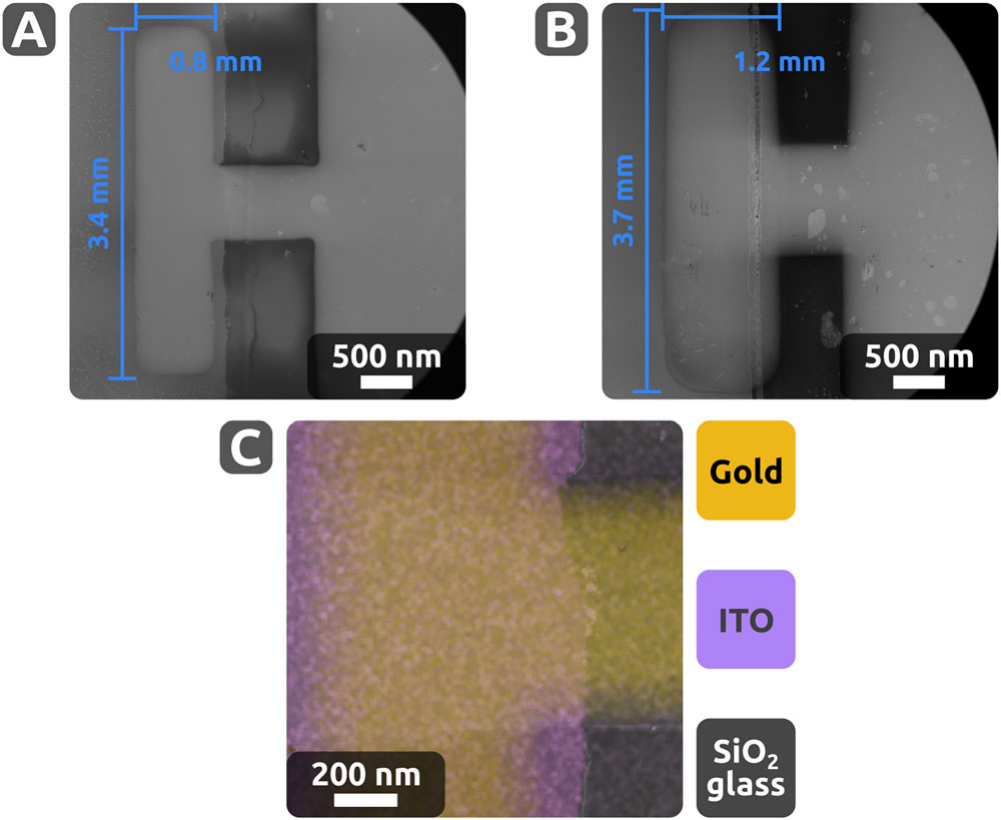
Device geometry. **A**, **B** SEM micrographs of the gold contacts defined by evaporation through a mask. They are positioned near the edge of a glass substrate bearing a surface on which the ITO layer has been removed by wet chemical etching. **A** and **B** present two nominally identical but distinct individual contacts and allow for a quantification of the positioning accuracy and tolerance in the contact dimensions. Differences are due to shadowing caused by an imperfectly adhering mask. **C** EDS mapping highlights the precision achieved for placing the connection bridge and active window sections of the gold contact as well as the edge of the ITO etching.

The geometric precision achieved by ALAM printing must be matched during the subsequent gold contact evaporation via conventional shadow masking. Figure 9 demonstrates that laser-cut PET foil masks deliver sufficiently tight tolerances (±0.2 mm) to address each cell without causing a short-circuit. The uncertainty is caused by bending of the PET foil, which introduces a shadow effect of slightly variable magnitude (±0.1 mm on each side). Despite this limitation, EDS mapping demonstrates that the edges of all relevant fields are sufficiently sharp for solar cell fabrication.

### Functional ALAM-printed cells

All ingredients prepared so far can now be combined to prepare a fully functional device. The ITO-coated glass slides are first etched locally as described in the previous section, then sputter-coated with 50 nm TiO_2_ and annealed (250 °C, 30 min, in air). ALAM is used to print six individual solar cells consisting of ZnS (30 passes, 1 nm), Sb_2_S_3_ (800/1200/1600 passes, 60/90/120 nm), and V_2_O_5_ (1600 passes, 34 nm). Annealing is performed after both the Sb_2_S_3_ deposition (300 °C, 2 min, under N_2_) and V_2_O_5_ (250 °C, 2 min, under N_2_). The ALAM-coated areas consist of a rectangle of ~2 mm × 19 mm dimensions obtained by printing eleven adjacent lines spaced by 0.2 mm nominally. Each such area contains three segments of distinct Sb_2_S_3_ layer thickness, demonstrating the prototyping capability of the approach—here, we explore Sb_2_S_3_ layers of 60 nm, 90 nm, and 120 nm. We chose the light absorber layer thickness to showcase the prototyping ability of our technology, given that it is the crucial parameter exploited experimentally to adjust the balance between the contradictory requirements of maximized light absorption and minimized loss of charge carriers by recombination on their way to the carrier selective materials^21^. Gold contacts are finally evaporated through the mask described above.

**Fig. 10:**
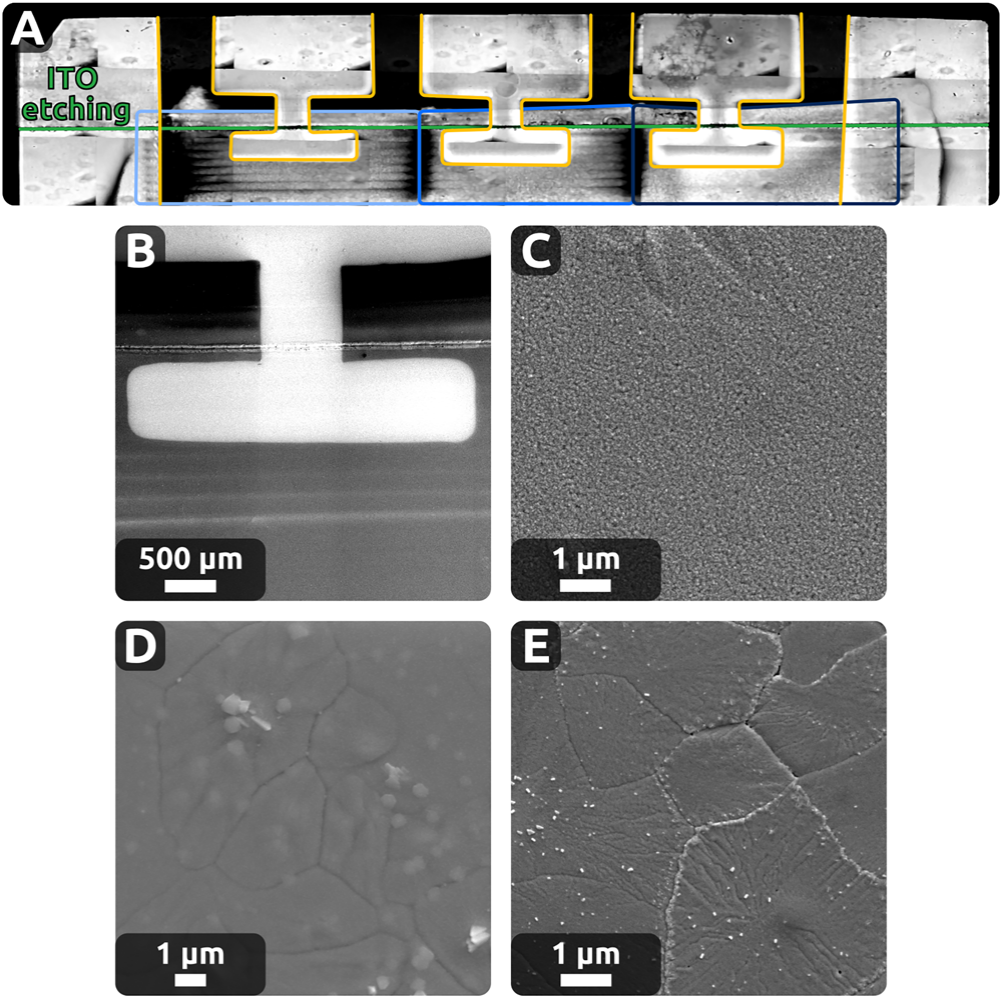
Functional solar cells. **A** Optical image of three solar cells fabricated by ALAM on one substrate. The precisely ALAM-printed features and evaporated gold contacts are visible. SEM micrographs (**B**–**E**) confirm the overall stack geometry (**B**) and, within it, the presence of morphological features observed previously for individual materials: homogeneous granularity of the top V_2_O_5_ layer (imaged with 3 kV acceleration voltage, **C**), crystalline Sb_2_S_3_/ZnS films with grain boundaries imaged underneath V_2_O_5_ (imaged under 15 kV, **D**) and in its absence (3 kV, **E**).

Optical images as well as SEM micrographs (Fig. 10A, B) evidence how the appearance of the structures reflects our design. SEM micrographs further reveal the surface morphology of the ALAM deposits: the fine roughness of the V_2_O_5_ layer (Fig. 10C) and the crystalline grain boundaries of the underlying Sb_2_S_3_ thin film (Fig. 10D, E).

**Fig. 11:**
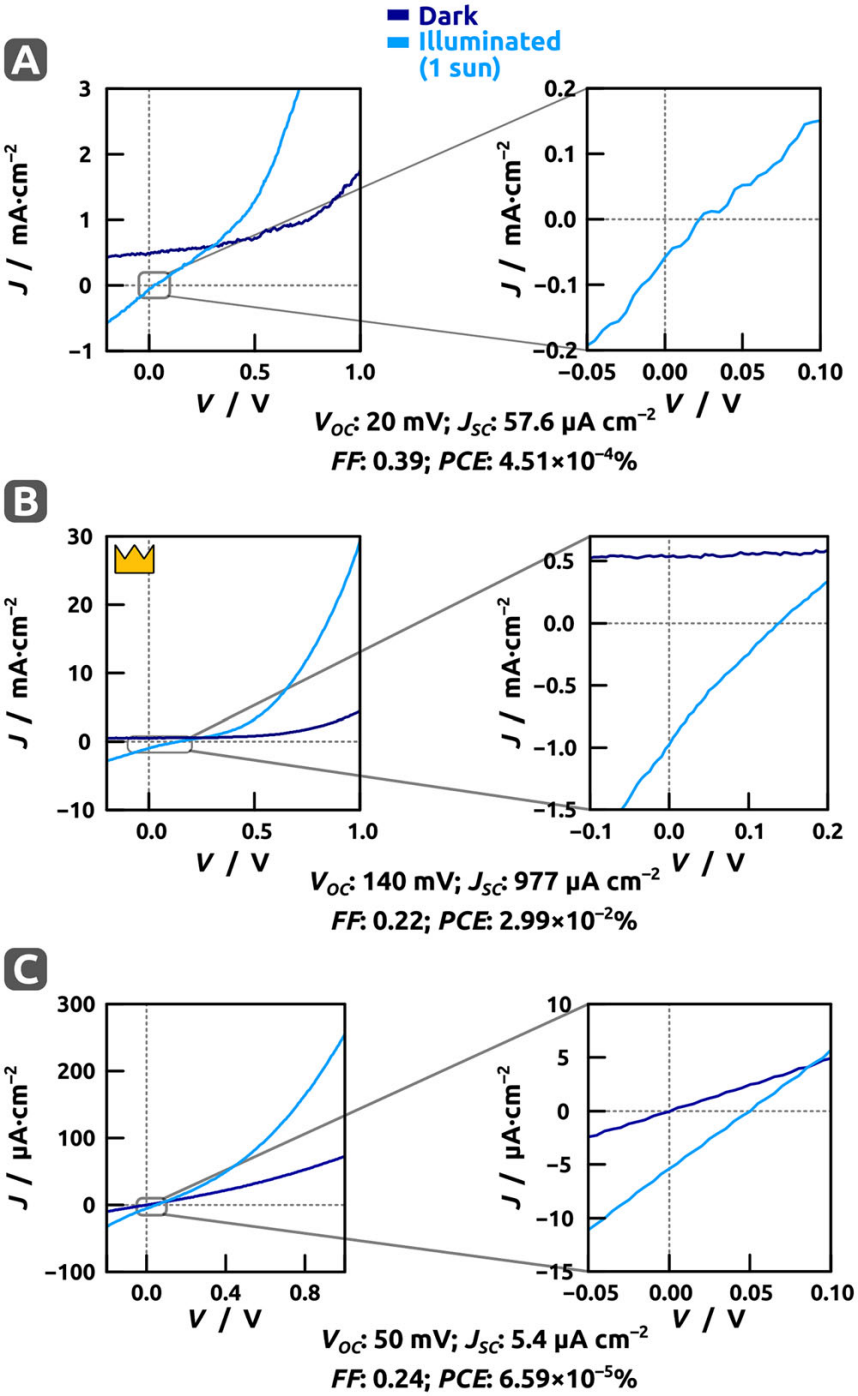
Functional performance. **A**–**C** *JV* measurements of three functional 3D-printed solar cells with 120 nm Sb_2_S_3_ layer thickness. All curves show the resemblance of diodes in dark conditions (royal blue) and under illumination (azure blue). These solar cells exhibit positive current in short-circuit in the dark as well as negative current in reverse bias conditions, indicating a low shunt resistance and the presence of (capacitive) leakage currents due to detrimental contacts. The *JV* curves visibly change under illumination (azure blue) and yield measurable photocurrents.

*JV* measurements (Fig. 11) establish the solar cell functionality and quantify performance. Five of the 18 stack units fabricated exhibit a rectifying behavior, and three of them clear solar cell characteristics with photocurrent and photovoltage under illumination. The occurrence of short-circuits is expected on the basis of the ZnO particles observed after ZnS deposition: they protrude vertically and increase the risk of direct contact between ZnO and V_2_O_5_. Here, the thickest Sb_2_S_3_ layer (120 nm) is needed to mitigate partly the effect of the ZnO particles by shutting down at least some of the short-circuits limiting *V*_*OC*_, with *V*_*OC*_ ranging between 20 and 140 mV and *J*_*SC*_ in the μA·cm^–2^ range. The very low current densities achieved here are related to the low fill factors, which are themselves due to the long lateral transport distances. In combination with the small active surface area and the low absolute values of the currents generated, the Ohmic losses are therefore significant. Additionally, the physical alignments of individual layers within the stack may not be perfect. Finally, the 120 nm thick Sb_2_S_3_ layer is necessary for avoiding short-circuits but thicker than what we have found to be optimal in a previous study^21^. Accordingly, the champion solar cell shows a *PCE* of only 0.03%. We note that this number does not fare so poorly when compared to the only example available so far in the openly accessible literature of what has been described as a “nearly all-ALD CIGS-type solar cell” (*J*_*SC*_ ≈ 500 μA cm^–2^, *V*_*OC*_ ≈ 80 mV, *FF* ≈ 0.5, *PCE* ≈ 0.02%)^63^.

These numbers are by no means on par with state-of-the-art photovoltaics reliant on Sb chalcogenides, and both performance and reliability must, in the future, be drastically improved. Most crucially, the ZnO particles must be eliminated using more elevated ZnS deposition temperatures, in order to avoid short-circuits. This simple proof-of-concept fabrication represents the first ever demonstration of solar cells 3D-printed with nanometer precision. We envision that this novel processing method will enable future optimization of photovoltaics that is not only faster but also significantly more efficient in both material usage and energy input. Further topics of relevance to be investigated in the future may pertain to the possibility that the imperfections of ALD chemistry yield organic residues in the films. Their presence strongly depends on the precursors chosen and the processing temperature^64–66^, and it would of course have a significant effect on the semiconductor properties.

## Conclusions

This study demonstrated that ALAM/DALP^®^ can be successfully employed to deposit thin films of Sb_2_S_3_, ZnS, and V_2_O_5_ in a 3D-printed manner with quality and layer thickness precision of conventional chamber-based ALD.

V_2_O_5_ ALAM relies on the metal-organic precursor (^*i*^PrO)_3_VO combined with water and resulting in ~350 μm-wide, continuous printed lines with a smooth, homogenous surface morphology, high compositional purity and crystalline structure after annealing.

ZnS printing is achieved by using the metal-organic precursor Zn(DMP)_2_ with H_2_S, yielding crystalline, smooth, and homogenous ZnS base layers on ~450 μm-wide defined lines. Additional ZnO agglomerates are caused by the limited substrate temperature achievable in the current prototype.

Sb_2_S_3_ thin lines are obtained from (Me_2_N)_3_Sb reacting with H_2_S. The as-printed ~500 μm-wide lines show high compositional purity but exhibit strong island growth and pinhole formation on metal oxide substrates, with additional dewetting of the thin films after annealing on Si(100) wafers. Introducing a ~1 nm thin ZnS adhesion layer beforehand prevents these undesirable effects. Annealed ALAM-printed ZnS / Sb_2_S_3_ structures exhibit a high crystallinity with continuous, smooth, and homogenous morphology. A limitation of the currently possible ZnS process is related to the temperatures achievable and the amount of residual water vapor present in the system. The presence of H_2_O has a significant, deleterious effect on the film morphology, adding large nodules to an otherwise smooth and continuous film. These structural imperfections lead to short-circuits which in the specific conditions used here have prevented us from building cells with optimal Sb_2_S_3_ layer thickness. This limitation, however, is a rather trivial one which will be addressed in a future overhaul of the ALAM tool.

A similar investigation could be performed by classical ALD. Exploiting ALAM instead offers several distinct advantages. Firstly, the amount of precursor consumed for this study is reduced by at least a factor 1000^46^. Secondly, the rapidity with which a process can be established (GPC determination) is incommensurable. Third, optimization of devices can be carried out in one deposition run on one substrate—a feat that is not accessible using classical blanket deposition techniques available so far.

The direct patterning capability of ALAM enables the integration of these individual semiconductors and the design of flexibly configurable small-scale arrays of functional solar cells on individual ITO/glass substrates. This study delivers a glimpse of the high throughput and prototyping capabilities of ALAM (and more generally, DALP^®^). With the early tool prototype used for this study, the total 3000 or so passes required for depositing all three semiconductors of one cell (corresponding to about 12,000 individual line passes for the whole area) are performed within 1 h. Aligning the lines and raster patterns used for the distinct layers of the semiconductor stack has been manual on the prototype tool. It will be upgraded to an automatized system in the near future, enabling sub-micron positioning reproducibility. The recent commercialization of a DALP^®^ reactor will ensure further time efficiency gains and improved reliability, thereby facilitating prompt acceptance of this technology.

## Experimental section

### Preparative methods

#### Chemicals and materials

The following materials were used: vanadium(V) oxytriisopropoxide (OV(OCH(CH_3_)_2_)_3_, 98%, *abcr*), bis(3-dimethylaminopropyl)zinc (Zn(DMP)_2_, synthesized)^67^, tris(dimethylamido)antimony(III) ([(H_3_C)_2_N]_3_Sb), ≥99%, *Sigma Aldrich* and *abcr*), water (*Milli-Q*^*®*^), hydrogen sulfide (H_2_S, 3% in N_2_, *Air Liquide*), Ar (N5.0, *Air Liquide*), N_2_ (N5.0, *Linde Germany*), Si(100) wafers (native oxide, *Silicon Materials Inc*.), ITO-coated glass substrates (≤10 Ω/sq., *Techinstro*), Hellmanex III (2% in *Milli-Q*^*®*^ water, *Hellma Analytics*), isopropanol (≥98% technical, *VWR*), acetone (≥99% technical, *VWR*), hydrochloric acid (HCl, 2 M, lab grade, *thermo fisher*), DALP^®^ nozzles (*Atlant3D*), Au (99.99% pellets, *m&k GmbH*), TiO_2_ target (99.99%), Si reference solar cell (*Newport*).

#### ALAM depositions

ALAM depositions were carried out using a DALP^®^ reactor prototype under atmospheric pressure of constantly purged N_2_ gas. Precursors and reactants were stored in separate bubblers connected to the DALP^®^ system. For vanadium(V) oxide ALAM, vanadium(V) oxytriisopropoxide was maintained at 50 °C with Ar serving as the carrier gas at a flow rate of 12 sccm. H_2_O was used as a reactant at room temperature (~23 °C) with N_2_ as the carrier gas at a flow rate of 30 sccm. Precursor and reactant vapors were transported through separate lines heated at 70 °C. The DALP^®^ nozzle system was maintained at 90 °C with an additional Ar flow of 600 sccm to ensure separation of the precursor/reactant channels. The substrate temperature was fixed at 150 °C. For zinc(II) sulfide ALAM, bis(3-dimethylaminopropyl)zinc was maintained at 65 °C with Ar as carrier gas at a flow rate of 12 sccm. H_2_S was used as a reactant at room temperature (~23 °C) at a flow rate of 30 sccm. Precursor and reactant vapors were transported through separate lines heated at 90 °C. The ALAM | DALP^®^ nozzle system was maintained at 110 °C with an additional Ar flow of 600 sccm to ensure separation of the precursor/reactant channels. The substrate temperature was fixed at 180 °C. For antimony(III) sulfide ALAM, tris(dimethylamido)antimony(III) was maintained at –3 °C with Ar as carrier gas at a flow rate of 12 sccm. H_2_S was used as a reactant at room temperature (~23 °C) at a flow rate of 30 sccm. Precursor and reactant vapors were transported through separate lines heated at 80 °C. The ALAM nozzle system was maintained at 130 °C with an additional Ar flow of 600 sccm to ensure separation of the precursor/reactant channels. The substrate temperature was fixed at 80 °C. Direct patterning was achieved by moving the substrate with a motorized stage at a speed of 200 mm/s and acceleration of 2000 mm/s^2^. The substrate was positioned visually as close as possible without contact, and the distance was kept constant throughout all prints.

### Solar cell preparation and fabrication

ITO glass substrates were completely covered with *Kapton*^*®*^ tape and subsequently cut using a laser cutter to obtain the ITO etching regions. ITO etching was performed with Zn powder/2 M HCl for 15 min. The *Kapton*^*®*^ tape was removed and the ITO glass substrate was subsequently cleaned by ultrasonication in 2% Hellmanex III solution, Isopropanol, Acetone, and *Milli-Q*^*®*^ water. As a final step, the substrates were treated with UV-ozone for 30 mins.

Subsequently, ~50 nm TiO_2_ from a TiO_2_ target (99.99%) was sputtered in RF mode on the cleaned ITO / glass substrates at a power density of 2.5 W cm^–2^ and Ar flow of 5 sccm with a base pressure of 1.3 × 10^–4^ Pa and working pressure of 4.3 Pa in a *Torr International Inc*. CRC 622 sputter coater. The TiO_2_ sputtered ITO glass substrates were subsequently annealed at 250 °C for 30 min in air.

After ZnS and Sb_2_S_3_ ALAM, the samples were annealed at 300 °C for 2 min under N_2_ atmosphere. After V_2_O_5_ ALAM printing, they were annealed again at 250 °C for 2 min under N_2_. As a last step, 80 nm thick gold back contacts were thermally evaporated using *a* Covap PVD instrument by *Ångstrom Engineering Inc*. from Au pellets (99.99%). A custom-built plastic foil mask was prepared via laser cutting, defining the active window size to ~0.5 × 3 mm^2^.

### Characterization methods

Imaging ellipsometry measurements were performed using an *Accurion EP-4 Ellipsometer* with an *Omicron LED Hub* providing six distinct wavelengths (384.9 nm, 447.2 nm, 522.7 nm, 595.4 nm, 657.7 nm and 840.6 nm). Angle of incidence and view were kept at 60.08°. All measurements were conducted with EP4Control 23.4.11.1 software from *Accurion*. Each ALAM print was scanned at all six wavelengths through automatic consecutive scans following a pre-programmed scanning pattern, with full automation of focusing and alignment at each step of the scan. The measured scans were stitched and analyzed with DataStudio 23.4.5.2 software from *Accurion*. Model calculations were performed using the EP4Model 23.4.4 software from *Accurion*. A self-calibrated Tauc-Lorentz function was used for all prints as mathematical models. To derive reliable mathematical models and obtain layer thicknesses, optical data were extracted after initial scanning with DataStudio 23.4.5.2 software. Separate datasets were generated: one for the optical properties of the underlying Si wafer (used for calibration) and others for each ALAM print segment. Clustering was performed to reduce computation time.

Optical microscopy was performed using the Imaging Ellipsometer’s built-in camera with 5-times magnification at a wavelength of 447.2 nm and image scanning/stitching.

Scanning electron microscopy was performed using a *Jeol JSM-F100* at an acceleration voltage of 3 kV and a working distance of 10 mm with secondary electron detector. Energy-dispersive X-ray spectroscopy (EDS) was carried out on the same instrument with an acceleration voltage between 5 kV and 15 kV. EDS spectra or maps were analyzed with SEM Center software from *Jeol*.

Grazing-incidence X-ray diffraction was performed on a *Bruker D8 Advance diffractometer* employing Cu Kα radiation (λ = 0.15406 nm) with a slit width of 0.1 mm under an incident angle of 0.5°. The 2*θ* measurements were conducted with 0.02° step size and 25 s measurement duration per step. The Crystallographic Open Database (COD) was used to assign expected diffractions.

Atomic force microscopy was performed on a *Bruker NanoMan VS* AFM in tapping mode using an OTESPA silicon tip with a spring constant of 26 N/m. Free amplitude was 1000 mV with a setpoint of 60–80%. Surface morphology was measured over a 1 × 1 µm^2^ area at a resolution of 1024 × 1024 samples per line, a scan rate of 0.5 Hz and tip velocity of 3 µm/s. Data and image processing was performed with Gwyddion 2.69 software.

Profilometry was performed using a *Bruker Dektak XT* Profilometer with a 20 s/600 µm scan and stylus force *(sic)* of 3 mg. Data was analyzed with Vision64 software from *Bruker*. Profile height was determined by plotting the height profile with Origin2025 from *OriginLabs*.

Current density - voltage (*JV*) measurements were performed under a solar simulator employing a Xenon lamp calibrated to 1.5 AM with 100 mW cm^–2^ illumination using a Si reference cell. Solar cells were connected using a Gamry Reference 600 potentiostat. Measurement sweep range was –1.1 V to +1.1 V with a step of 5 mV and scanning speed of 30 mV s^–1^. Datasets were plotted with Origin2025 from *OriginLabs*.

## Data Availability

The data (raw and analyzed) presented in the figures is available on the open access platform Zenodo under the 10.5281/zenodo.19473184.
